# Effect of Clinical, Endoscopic, Radiological Findings, and Complications on Survival in Patients with Primary Gastrointestinal Lymphoma

**DOI:** 10.5152/tjg.2022.211003

**Published:** 2022-11-01

**Authors:** Murat Erkut, Nergiz Erkut, Özlen Bektaş, Sami Fidan, Arif Mansur Coşar, Mehmet Sönmez

**Affiliations:** 1 Department of Gastroenterology, Karadeniz Technical University Faculty of Medicine, Trabzon; 2 Department of Hematology, Karadeniz Technical University Faculty of Medicine, Trabzon

**Keywords:** Endoscopic finding, complication, primary gastrointestinal lymphoma, prognosis, radiological finding

## Abstract

**Background:**

The purpose of this study was to evaluate the clinical, endoscopic, and radiological characteristics, complications, survival outcomes, and prognostic factors of patients with primary gastrointestinal lymphoma.

**Methods:**

This study retrospectively analyzed the demographic, laboratory, endoscopic, and radiological characteristics and treatment outcomes of 43 patients with newly diagnosed primary gastrointestinal lymphoma.

**Results:**

The median age was 62 years (range: 26-83). The primary lesion location was the gastric in 33 (77%) patients and the intestinal in 10 (23%) patients. The most common lesions were the corpus (33%) and corpus + antrum (24%) in primary gastric lymphoma and the ileum (60%) in primary intestinal lymphoma. The most common endoscopic findings were diffuse infiltrative lesion (23%) and mass-forming (33%), while the most common computed tomography finding was wall thickening (53%). Wall thickening and mass-forming at computed tomography were greater in primary intestinal lymphoma than in primary gastric lymphoma (*P* = .034). Complications were observed in 9 (21%) patients and 13 (31%) patients who underwent surgery. Complication and surgery rates were higher in primary intestinal lymphoma than in primary gastric lymphoma (*P* = .003 and *P* = .014, respectively). Five-year overall survival and 5-year event-free survival rates were 75% and 72%, respectively. Univariate analysis showed that intestinal involvement, advanced clinical stage, a high International Prognostic Index score, mass-forming and wall thickening at computed tomography, extranodal involvement, and complication were found to adversely affect survival. Multivariate analysis revealed that intestinal involvement and a high International Prognostic Index score were independent prognostic factors for overall survival and event-free survival.

**Conclusion:**

Patients with primary gastrointestinal lymphoma with intestinal involvement and high International Prognostic Index score should be followed closely.

## Introduction

Primary gastrointestinal lymphoma (PGIL) is a malignancy derived from the gastrointestinal tract and spreads primarily through neighboring lymph node invasion.^1,2^ It constitutes 30%-40% of extranodal lymphomas and 1%-4% of malignant gastrointestinal tract tumors.^3,4^ Monoclonal proliferation developing as a result of antigenic stimulation and recurring inflammation plays a key role in the pathogenesis.^1,2^ Some possible risk factors such as *Helicobacter pylori* (HP), human immunodeficiency virus, and Epstein–Barr virus infection, celiac disease, autoimmune diseases, immunosuppressive drugs, and inflammatory bowel diseases are involved in the etiology.^1,5^

Primary gastrointestinal lymphoma is most frequently seen in the gastric, followed by ileocecal region, small bowel, and colon in the gastrointestinal tract.^6,7^ Histologically, diffuse large B-cell lymphoma (DLBCL) and mucosa-associated lymphoid tissue (MALT) lymphoma are most common, while follicular lymphoma (FL), mantle cell lymphoma (MCL), Burkitt lymphoma (BL), enteropathy-associated T-cell lymphoma, and post-transplant lymphoproliferative disease are less common.^8,9^ Endoscopic biopsies from the region with lymphoma involvement in the gastrointestinal tract are required for diagnosis.^10^ Computed tomography (CT), magnetic resonance imaging (MRI), endoscopic ultrasonography, or ^18^F-fluorodeoxyglucose positron emission tomography are used to assess the spread of the disease.^11^ Treatment options include HP eradication therapy, immunotherapy, chemotherapy, surgery, and radiotherapy, although the optimal treatment for PGIL still remains controversial.^12^ Life-threatening complications such as perforation, bleeding, and obstruction can also be seen during the course of the disease.^13^

Although the medical literature contains studies concerning PGIL, the number of studies evaluating the relationship between endoscopic and radiological findings, complications, and survival is limited. The purpose of this study was to evaluate the clinical, endoscopic, and radiological characteristics, complications, survival outcomes, and prognostic factors of patients with PGIL.

## MATERIALS AND METHODS

### Patient Selection

This study was carried out on patients with newly diagnosed PGIL between January 2006 and August 2020 at the Karadeniz Technical University Medical Faculty. Diagnosis of PGIL was based on Dawson standards.^14^ Patients younger than 18 years of age were excluded from this study. Demographic data, laboratory, endoscopic, and radiological findings, complications, treatments, and responses were collected retrospectively from our hospital’s electronic data record system. Since this article is a retrospective study, informed consent was not obtained from the patients. Approval for the study was granted by the ethic committee of Karadeniz Technical University Medical Faculty under protocol no. 2021/92.

### Diagnostic Procedure

Esophagogastroduodenoscopy, ileocolonoscopy, or surgical resection when clinical presentations did not permit endoscopic procedures were performed based on the sites of lymphoma involvement in the gastrointestinal tract. Preparates from tissue samples obtained from endoscopic biopsy and/or surgical resection were stained with hematoxylin-eosin and were assessed using immunohistochemical methods (CD20 and CD3 routinely for all patients and CD5, CD10, CD23, CD15, CD30, terminal deoxynucleotidyl transferase [tdt], cyclin D1, Bcl-2, Bcl-6, c-myc, p53, Ki-67, and immunoglobulin light chain [κ, λ] for selected patients). In line with the World Health Organization 2016 Lymphoma Classification, MALT lymphoma, FL, and MCL were classified as low-grade lymphomas and DLBCL, BL, lymphoblastic lymphoma, and T-cell lymphoma as high-grade lymphomas.^15^

Pathological lesions detected during endoscopic procedures were defined as superficial, diffuse infiltrative, ulcer, or mass-forming. Computed tomography findings were defined as normal, wall thickening, or mass-forming.

### Staging and Prognosis

Primary gastrointestinal lymphoma staging was performed using the Lugano International Conference classification based on physical examination, laboratory findings (complete blood count, lactate dehydrogenase [LDH], albumin, erythrocyte sedimentation rate [ESR]), imaging techniques (posteroanterior x-ray and neck-thoracic-abdominal-pelvic CT), and bone marrow biopsy.^16^ Stage I and stage II were defined as early stage and stage IV as advanced stage. Patients were also classified as low risk (scores 0-1), low-moderate risk (score 2), moderate-high risk (score 3), or high risk (scores 4-5) based on the International Prognostic Index (IPI).^17^

### Treatment and Response Evaluation

The patients received the following chemotherapy protocols: R-CHOP (rituximab-cyclophosphamide, doxorubicin, vincristine, and prednisone) for DLBCL, HyperCVAD (hyperfractionated cyclophosphamide, vincristine, doxorubicin, and dexamethasone) for BL, R-CHOP, R-COP (rituximab-cyclophosphamide, doxorubicin, and prednisone), and R alone for MALT lymphoma, R-CHOP for MCL. Patients with MALT lymphoma also received HP eradication therapy. Patients with relapse and refractory were given high-dose chemotherapy. Surgical procedures were performed for diagnosis or complications. Responses to treatment were defined using the Lugano classification as complete remission (CR), partial remission (PR), stable disease (SD), or progressive disease (PD).^18^

### Statistical Analysis

Overall survival (OS) was defined as the time between diagnosis and death from any cause or the last control examination. Event-free survival (EFS) was defined as the time between diagnosis and disease progression, disease recurrence, death from any cause, or the last control examination. The Kaplan–Meier method was calculated for OS and EFS analyses. The log-rank test was used to perform a univariate analysis. Multivariate analysis with the Cox proportional hazards model was carried out to analyze variables affecting prognosis. The chi-square test was used for categorical and ordinal variables. The Mann–Whitney *U *test was employed for non-parametric variables. Student’s *t*-test was used for quantitative variables. *P* < .05 was considered statistically significant. All statistical analyses were performed in Statistical Software Package for the Social Sciences version 23 software (IBM Corp.; Armonk, NY, USA).

## Results

### Patient Characteristics

The median age of the 43 patients enrolled in the study was 62 years (range: 26-83). Ten (23%) were women and 33 (77%) were men. Mean hemoglobin level was 12.2 (±2.4) g/dL, median white blood cell count was 8 (range: 3.9-18.5) × 10^9^/L, median platelet count was 281 (range: 52-651) × 10^9^/L, median ESR level was 31 (range: 2-120) mm/h, median LDH level was 227 (range: 143-1644) U/L, and median albumin level was 3.9 (range: 2-5) mg/dL.

Histological type was DLBCL in 32 (74%) patients, MALT lymphoma in 7 (16%) patients, MCL in 2 (5%) patients, and BL in 2 (5%) patients. Eighteen (42%) patients were stage I, 14 (33%) stage II, and 11 (25%) stage IV. According to IPI scores, 23 (54%) patients were in the low risk, 9 (21%) patients in the low-moderate risk, 4 (9%) patients in the high-moderate risk, and 7 (16%) patients in the high-risk group. Extranodal involvement was present in 10 (23%) patients.

The primary lesion location was the gastric in 33 (77%) patients and the intestinal in 10 (23%) patients. Primary gastric lymphoma (PGL) lesions were 11 (33%) in the corpus, 5 (15%) in the antrum, 5 (15%) in the cardia/fundus, 8 (24%) in the corpus + antrum, and 4 (12%) in the cardia/fundus + corpus region. Primary intestinal lymphoma (PIL) lesions were 6 (60%) in the ileum, 2 (20%) in the duodenum, and 2 (20%) in the colon region.

Complications were observed in 9 (21%) patients including bleeding in 3 (7%), obstruction in 3 (7%), perforation in 2 (5%), and bleeding + perforation in 1 (2%).

Thirty-eight (88%) patients were treated with chemotherapy and 2 (5%) patients were applied immunotherapy alone. Eight (19%) patients received HP eradication (20 patients with PGL were evaluated with HP diagnostic tests). Eight (19%) patients underwent surgery including 5 (12%) patients with emergency surgery. One (%2) patient was applied radiotherapy. Three (7%) patients were treated with high-dose chemotherapy. The patients’ characteristics are shown in Table 1.

### Endoscopy and Computed Tomography Findings

Macroscopically, endoscopic images viewed superficial lesions in 2 (5%) patients, diffuse infiltrative lesions in 10 (23%) patients, ulcer in 8 (19%) patients, and mass-forming in 14 (33%) patients. Endoscopy reports were unavailable for 9 (21%) patients undergoing emergency surgery due to presentation findings and/or undergoing diagnostic procedures outside our hospital. Computed tomography detected wall thickening in 22 (51%) patients and mass-forming in 7 (16%) patients, while no abnormality found in 14 (33%) patients (Table 2).

**The Relationship Between the Primary Lesion Location and Clinical, Endoscopic, and Radiological Findings**

No difference was determined between PGL and PIL in terms of age, gender, or laboratory tests. Eight (24%) patients were low-grade lymphoma and 25 (76%) patients were high-grade in PGL, while 1 (10%) patient was low-grade lymphoma and 9 (90%) patients were high-grade in PIL. Twenty-seven (82%) patients were early stage and 6 (18%) patients were advanced stage in PGL, while 5 (50%) patients were early stage and 5 (50%) patients were advanced stage PIL. Twenty-seven (82%) patients were in the ≤2 IPI score and 6 (18%) patients were in the ≥3 IPI score in PGL, while 5 (50%) patients were in the ≤2 IPI score and 5 (50%) patients were in the ≥3 IPI score in PIL. Extranodal involvement was present in 5 (15%) patients in PGL and in 5 (50%) patients in PIL. No statistically significant difference was observed between PGL and PIL in terms of lymphoma grade, stage, IPI score, or extranodal involvement. Complications developed in 3 (9%) patients in PGL and in 6 (60%) patients in PIL. Surgery was performed on 3 (9%) patients in PGL and on 5 (50%) patients in PIL. Complication and surgery rates were significantly higher in PIL than PGL (*P* = .003 and *P* = .014, respectively) (Table 3).

Endoscopic images were viewed superficial, ulcer, or diffuse infiltrative lesion in 18 (67%) patients and mass-forming in 9 (33%) patients in PGL, while superficial, ulcer, or diffuse infiltrative lesion was seen in 2 (29%) patients and mass-forming in 5 (71%) patients in PIL. But endoscopic findings were not significantly different between the 2 groups.

Computed tomography detected wall thickening or mass-forming in 19 (58%) patients in PGL, while no abnormal findings showed in 14 (42%) in PGL. Wall thickening or mass-forming were detected in 10 (100%) patients in PIL. Computed tomography findings of wall thickening or mass-forming were significantly more common in PIL than in PGL (*P* = .034).

### Treatment Response, Survival Analysis, and Prognostic Factors

Treatment response was CR in 36 (84%) patients, PR in 2 (5%) patients, and PD in 3 (7%) patients. Early mortality occurred in 2 (5%) patients. Relapse developed in 4 (9%) patients. Eleven (26%) patients died. Overall survival and EFS times were 95 months (95% CI: 79-111 months) and 94 months (95% CI: 78-111 months), respectively. Five-year OS and EFS rates were 75% and 72%, respectively.

**Figure 1.**
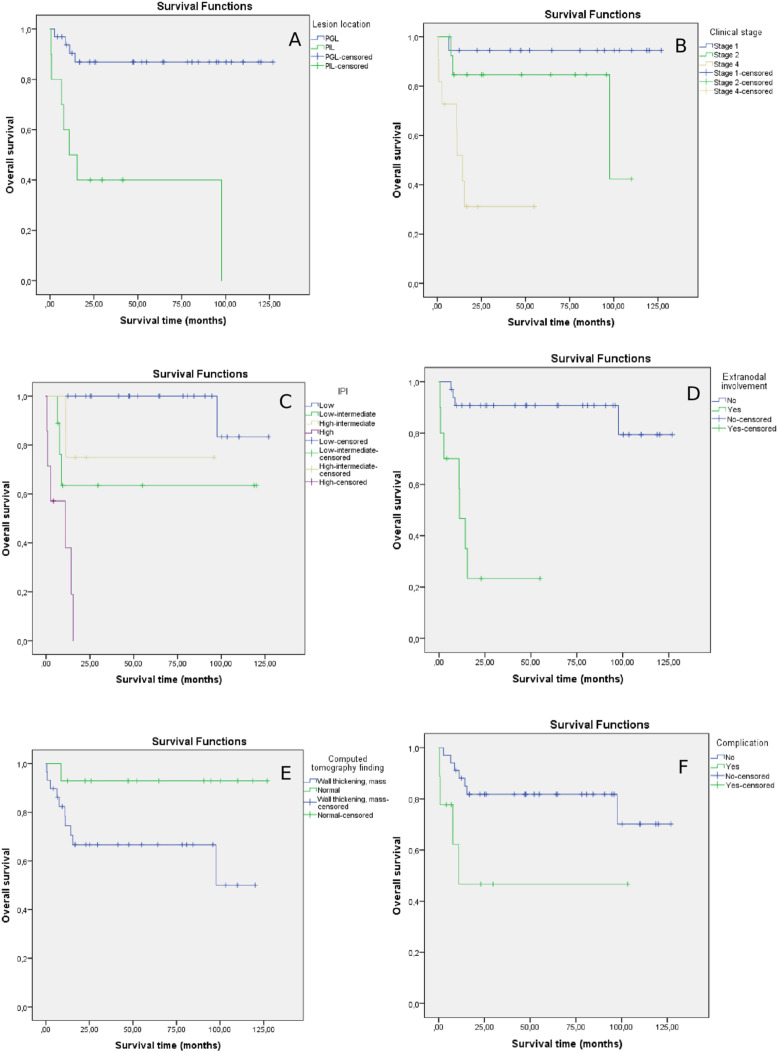
Univariate analysis: OS (A) according to the lesion location (*P* < .001). OS (B) according to the clinical stage (*P *< .001). OS (C) according to IPI (*P* < .001). OS (D) according to the presence of extranodal involvement (*P *< .001). OS (E) according to CT finding (*P* = .047). OS (F) according to the presence of complication (*P* = .027).

No statistically significant differences in OS and EFS times were observed between female and male, <60 and ≥60 years, or high-grade and low-grade patients. Overall survival and EFS times were longer in patients with gastric location than in patients with intestinal location (OS 112 months [95% CI: 97-126], 43 months [95% CI: 73-113], *P* < .001; EFS 112 months [95% CI: 97-126], 23 months [95% CI: 6-39], *P* < .001, respectively). Overall survival and EFS times were longer in stage I and II patients than in stage IV patients (OS 120 months [95% CI: 107-133], 89 months [95% CI: 69-109], 23 months [95% CI: 9-36], *P* < .001, *P* = .012; EFS 120 months [95% CI: 106-133], 86 months [95% CI: 63-109], 20 months [95% CI: 5-35], *P* < .001, *P* = .008, respectively). Overall survival and EFS times were longer in IPI score low-risk patients than in low-moderate risk, high-moderate risk, and high-risk patients (OS 122 months [95% CI: 113-131], 79 months [95% CI: 42-116], 75 months [95% CI: 39-111], 8 months [95% CI: 3-13], *P* = .018, *P* = .016, *P* < .001; EFS 121 months [95% CI: 110-132], 79 months [95% CI: 42-117], 74 months [95% CI: 36-111], 4 months [95% CI: 0-7], *P *= .014, *P* = .075, *P* < .001, respectively). Additionally, OS and EFS times were shorter in IPI score high-risk patients than in low-risk, low-moderate risk, and high-moderate risk patients (OS: *P* < .001, *P* = .035, *P* = .028; EFS: *P* < .001, *P* = .012, *P* = .023, respectively). No significant difference was observed between IPI score low-moderate risk and high-moderate risk patients. Overall survival and EFS times were shorter in patients with extranodal involvement than in those without extranodal involvement (19 months [95% CI: 6-33], 112 months [95% CI: 99-126], *P* < .001; EFS 16 months [95% CI: 2-31], 111 months [95% CI: 97-126], *P* < .001, respectively). There was no significant difference among patients with endoscopic findings of superficial, ulcer, or diffuse infiltrative lesions and those with mass-forming in terms of OS and EFS times. Overall survival and EFS times were shorter in patients with CT findings of wall thickening or mass-forming than in patients with CT findings of normal (79 months [95% CI: 59-99], 118 months [95% CI: 103-134], *P* = .047; EFS 78 months [95% CI: 57-99], 119 months [95% CI: 103-134], *P* = .050, respectively). Overall survival and EFS times were shorter in patients with complications than in those without complications (51 months [95% CI: 15-87], 102 months [95% CI 86-118], *P* = .027; EFS 56 months [95% CI: 21-90], 101 months [95% CI: 85-118], *P* = .041, respectively). No significant difference was observed in OS and EFS times between patients who underwent surgery and those without surgery (Table 4, Figure 1).

Significant independent prognostic factors for better OS and EFS were primary lesion location and IPI score at multivariate analysis. The risk of mortality in patients with PIL was approximately 8.5 times higher than in patients with PGL. Also, the risk of mortality was approximately 9.5 times higher in patients with high IPI scores than low IPI scores (Table 4).

## Discussion

Primary gastrointestinal lymphoma is a rare malignancy capable of emerging in different regions of the gastrointestinal system. Gastric involvement is the most common, with a reported frequency of 66.7%-76.1%.^4,19,20^ However, in a study from China, Li et al^13^ reported that the most common site of involvement was the intestinal region at 55.1%, with gastric involvement being seen at a rate of 38.5%. Intestinal lymphoma is more common in Middle Eastern countries, where the prevalence of immunoproliferative small intestinal disease is high.^21^ This variation between societies may be due to geographical changes in infections, autoimmune diseases, and environmental factors.^19^ In the present study, similarly to Western societies, gastric involvement was detected at a rate of 77% and intestinal involvement at 33%.

While the most frequently involved region in patients with PGL is the corpus or the fundus, in patients with PIL is the ileum.^5^ Ge et al^22^ reported that the involvement sites in order of frequency in the gastric were 43.5% in the antrum, 34.8% in the corpus and 8.7% in the cardia-fundus. The involvement sites in the intestinal were 70.3% in the ileum, 16.2% in the jejunum, and 8.1% in the duodenum. Similarly in the present study, the majority of PGL lesions were in the corpus and antrum regions, while PIL lesions were largely in the ileum, findings compatible with the previous literature.

The most frequently seen histological type in PGL is MALT lymphoma, followed by DLBCL, while observed histological type in PIL are DLBCL, MCL, MALT lymphoma, FL, and T-cell lymphoma.^5^ On the other hand, a study from Turkey^23^ reported incidences of 87.5% for DLBCL and 8.9% for MALT lymphoma in the gastric. In the small intestine and colon, incidence rates for DLBCL are 45.5% and 12.5%, respectively. In the present study, consistent with this other study from Turkey, a higher rate of DLBCL was observed in the gastric. The histological type variation in patients diagnosed with PGL in Turkish society may be related to various environmental factors, particularly geographical factors and dietary habits. In a study of patients with PGIL, Nakamura et al^19^ reported that stage I and low-grade lymphoma were more dominant in patients with gastric involvement and stage II and high-grade lymphoma in those with intestinal involvement. No significant difference was observed in the present study between PGL and PIL in terms of either stage or histological grade. Additionally, no difference was observed in terms of IPI scores between gastric and intestinal involvement. The insignificant differences between the groups may be due to the low patient numbers.

Superficial, ulcer, diffuse infiltrative, and mass lesions can be seen on the endoscopic images of patients with PGIL. Wall thickening or mass is detected in 85% of cases at abdominal CT.^12^ In the present study, superficial, ulcer, or diffuse infiltrative lesions were more frequent on the endoscopic images from patients with PGL, while mass lesions were more common in patients with PIL, but the difference was not statistically significant. Wall thickening or mass-forming was more frequently detected at CT in patients with PIL than in those with PGL. Complications such as obstruction and perforation are more frequently seen in patients with PGIL presenting with mass lesions, especially those with intestinal involvement, and this increases surgical requirements. Li et al^13^ reported that complications were more common in patients with intestinal involvement compared to those with gastric involvement (28.6% and 10.8%, respectively). Complication development and surgery requirement rates were also higher in PIL compared to PGL in the present study. Surgery requirements associated with severe complications in patients with PGIL have been reported at less than 5%.^24^ In contrast to the previous literature, however, emergency surgery due to complication development was performed on 12% of patients enrolled in the present study. It should be remembered that the risk of complications, and therefore surgery requirements, may be higher in patients with PIL in particular, due to the bowel wall being thinner and the greater frequency of mass lesions.

Different survival rates associated with PGIL have been reported. Li et al^13^ reported 5-year OS and EFS rates of 56.4% and 49.3%, while Nakamura et al^19^ reported rates of 72% and 68%. Five-year OS and EFS rates in the present study, similar to the study of Nakamura et al^19^, were 75% and 72%, respectively. Nakaramu et al^19^ determined early stage, young age, gastric involvement, B-cell phenotype, and absence of B symptoms as good prognostic factors for OS. Gou et al^25^ identified male gender, absence of radical surgery, and T-cell phenotype as poor prognostic factors for OS in patients with PGIL. Chen et al^2^ described multiple area involvement and IPI ≥2 as poor risk factors for OS and progression-free survival in patients with gastrointestinal B-cell lymphoma. Li et al^13^ reported performance status, LDH levels, and histological type as independent prognostic risk factors in patients with PGIL. In the present study, primary lesion location, stage, IPI, extranodal involvement, mass and wall thickening at CT, and complications emerged as associated with survival at univariate analysis. But only primary lesion location and IPI are independent risk factors in the multivariate analysis.

The most important limitations of our study are that it is a retrospective study and the number of cases is low. Other limitations are the lack of endoscopy reports for some patients. In addition, it was also not possible to evaluate the HP eradication therapy results due to HP not having been diagnosed in some patients.

## Conclusions

The most frequent primary lesion location was gastric in patients with PGIL in this study. Abnormal CT findings, complications, and surgery requirements were more frequent in patients with intes­tinal involvement than in those with gastric involvement. Univariate analysis showed that intestinal involvement, advanced clinical stage, a high IPI score, mass-forming and wall thickening at CT, extranodal involvement, and complication were found to adversely affect survival. Multivariate analysis revealed that intestinal involve­ment and a high IPI score were independent prognostic factors for OS and EFS. Therefore, careful monitoring is recommended, especially in patients with high intestinal involvement and high IPI scores.

## Figures and Tables

**Table 1.** Characteristics of Patients with Primary Gastrointestinal Lymphoma

**Characteristics**	
Gender, n (%) Female Male	10 (23) 33 (77)
Age years, median (range)	62 (26-83)
Hb g/dL, mean (±SD)	12.2 (±2.4)
WBC ×10^9^/L, median (range)	8 (3.9-18.5)
Platelet ×10^9^/L, median (range)	281 (52-651)
ESR mm/h, median (range)	31 (2-120)
LDH U/L, median (range)	227 (143-1644)
Albumin mg/dL, median (range)	3.9 (2-5)
Histological type, n (%) DLBCL MALT lymphoma MCL BL	32 (74) 7 (16) 2 (5) 2 (5)
Primary lesion location, n (%) Gastric Cardia-fundus Corpus Antrum Cardia-fundus + corpus Corpus + antrum Intestinal Duodenum Ileum Colon	33 (77) 5 (15) 11 (33) 5 (15) 4 (12) 8 (24) 10 (23) 2 (20) 6 (60) 2 (20)
Stage, n (%) I II IV	18 (42) 14 (33) 11 (25)
IPI, n (%) Low risk Low-moderate risk High-moderate risk High risk	23 (54) 9 (21) 4 (9) 7 (16)
Extranodal involvement, n (%)	10 (23)
Complication, n (%) Hemorrhage Obstruction Perforation Hemorrhage + perforation	9 (21) 3 (7) 3 (7) 2 (5) 1 (2)
Treatment, n (%) Chemotherapy Immunotherapy alone HP eradication Surgery Emergency surgery Radiotherapy	38 (88) 2 (5) 8 (19) 8 (19) 5 (12) 1 (2)

Hb, hemoglobin; SD, standard deviation; WBC, white blood cell count; ESR, erythrocyte sedimentation rate; LDH, lactate dehydrogenase; DBBHL, diffuse large B-cell lymphoma; MALT, mucosa-associated lymphoid tissue; MCL, mantle cell lymphoma; BL, Burkitt lymphoma; IPI, international prognostic index; HP, *Helicobacter pylori*.

**Table 2.** Endoscopy and Computed Tomography Findings of Patients with Primary Gastrointestinal Lymphoma

**Findings**	**Total, n (%)**	**PGL, n (%)**	**PIL, n (%)**
Endoscopic Superficial Diffuse infiltrative Ulcer Mass-forming Unknown	2 (5) 10 (23) 8 (19) 14 (33) 9 (21)	2 (6) 9 (27) 7 (21) 9 (27) 6 (18)	0 (0) 1 (10) 1 (10) 5 (50) 3 (30)
CT Normal Wall thickening Mass-forming	14 (33) 22 (51) 7 (16)	14 (42) 16 (48) 3 (9)	0 (0) 6 (60) 4 (40)

PGL, primary gastric lymphoma; PIL, primary intestinal lymphoma; CT, computed tomography.

**Table 3.** Comparison of Primary Lesion Location of Patients with Primary Gastrointestinal Lymphoma

**Characteristics**	**n**	**PGL**	**PIL**	***P***
Gender, n (%) Female Male	10 33	8 (24) 25 (76)	2 (20) 8 (80)	.781
Age years, n (%) <60 ≥60	19 24	16 (49) 17 (51)	3 (30) 7 (70)	.504
Hb g/dL, mean (±SD)	33/10	12.6 (±2)	11.3 (±3.3)	.261
WBC ×10^9^/L, median (range)	33/10	8 (3.9-18.5)	7.9 (4.2-11.9)	.581
Platelet ×10^9^/L, median (range)	33/10	281 (121-651)	263 (52-455)	.702
ESR mm/h, median (range)	33/10	30 (3-120)	31.5 (2-79)	.966
LDH U/L, median (range)	33/10	218 (146-1512)	261 (143-1644)	.402
Albumin mg/dL, median (range)	33/10	3.9 (2.5-5)	3.8 (2-4.4)	.542
Endoscopic findings, n (%) Superficial/diffuse infiltrative/ulcer Mass-forming	20 14	18 (67) 9 (33)	2 (29) 5 (71)	.068
CT findings, n (%) Normal Wall thickening/mass-forming	14 29	14 (42) 19 (58)	0 (0) 10 (100)	.034*
Histological type, n (%) High grade Low grade	34 9	25 (76) 8 (24)	9 (90) 1 (10)	.332
Stage, n (%) Early stage Advanced stage	32 11	27 (82) 6 (18)	5 (50) 5 (50)	.108
IPI, n (%) ≤2 ≥3	32 11	27 (82) 6 (18)	5 (50) 5 (50)	.108
Extranodal involvement, n (%) Yes No	10 33	5 (15) 28 (85)	5 (50) 5 (50)	0.063
Complication, n (%) Yes No	9 34	3 (9) 30 (91)	6 (60) 4 (40)	.003*
Surgery, n (%) Yes No	8 35	3 (9) 30 (91)	5 (50) 5 (50)	.014*

PGL, primary gastric lymphoma; PIL, primary intestinal lymphoma; Hb, hemoglobin; WBC, white blood cell count; ESR, erythrocyte sedimentation rate; LDH, lactate dehydrogenase; CT, computed tomography; IPI, international prognostic index. *P < .05 was considered statistically significant.

**Table 4.** Univariate and Multivariate Analysis of Patients with Primary Gastrointestinal Lymphoma

**Univariate Characteristics**	**n**	**Mean OS, Months (95% CI)**	**5-Year OS Rate, %**	***P***	**Mean EFS, Months (95% CI)**	**5-Year EFS Rate, %**	***P***
Gender Female Male	10 33	102 (72-133) 88 (70-105)	80 74	.635	102 (70-133) 87 (68-105)	80 69	.660
Age, years <60 ≥60	19 24	108 (88-128) 81 (79-111)	84 68	.156	108 (87-128) 79 (57-102)	84 62	.177
Primary lesion location Gastric Intestinal	33 10	112 (97-126) 43 (73-113)	87 0	<.001*	112 (97-126) 23 (6-39)	87 0	<.001*
Histological type High grade Low grade	34 9	89 (72-106) 100 (70-129)	72 87	.732	89 (71-106) 101 (70-132)	73 72	.682
Stage I II IV	18 14 11	120 (107-133) 89 (69-109) 23 (9-36)	94 85 31	<.001*	120 (106-133) 86 (63-109) 20 (5-35)	94 70 32	<.001*
IPI Low risk Low-moderate risk High-medium risk High risk	23 9 4 7	122 (113-131) 79 (42-116) 75 (39-111) 8 (3-13)	100 63 75 0	<.001*	121 (110-132) 79 (42-117) 74 (36-111) 4 (0-7)	92 65 75 0	<.001*
Extranodal involvement Yes No	10 33	19 (6-33) 112 (99-126)	23 90	<.001*	16 (2-31 days) 111 (97-126)	25 85	<.001*
Endoscopic findings Superficial/diffuse infiltrative/ulcer Mass-forming	20 14	103 (81-124) 74 (46-102)	80 62	.202	102 (81-124) 71 (41-100)	80 52	.161
CT findings Normal Wall thickening/mass-forming	14 29	118 (103-134) 79 (59-99)	93 66	.047*	119 (103-134) 78 (57-99)	93 61	.050*
Complication Yes No	9 34	51 (15-87) 102 (86-118)	46 82	.027*	56 (21-90) 101 (85-118)	53 77	.041*
Surgery Yes No	8 35	66 (33-99) 98 (81-115)	62 78	.321	65 (31-99) 98 (80-115)	62 74	.304
**Multivariate** **Characteristics**	**HR [95% CI]**				***P***		
Gender Female Male	2.186 (0.344-13.873)				.407		
Age, years <60 ≥60	4.073 (0.805-20.612)				.090		
Primary lesion location Gastric Intestinal	8.439 (1.420-50.140)				.019*		
IPI Low risk Low-moderate risk High-moderate risk High risk	9.555 (1.773-51.485)				.009*		
Complication Yes No	0.567 (0.099-3.253)				.524		
CT findings Normal Wall thickening/mass-forming	1.015 (0.067-15.484)				.991		

The multivariate analysis model was made with gender, age, primary lesion location, IPI, complication, and CT findings. Omnibus tests of model coefficients: −2 log likelihood, 53.925, chi-square, 27.179, *P*: .000.

*P < .05 was considered statistically significant.

OS, overall survival; EFS, event-free survival; IPI, international prognostic index; CT, computed tomography; HR, hazard ratio.
